# Comparative thoracic radiography in healthy and tuberculosis-positive sun bears (*Helarctos malayanus*)

**DOI:** 10.3389/fvets.2024.1460140

**Published:** 2025-01-06

**Authors:** Kirsty Officer, Natalie Webster, Alana J. Rosenblatt, Phorn Sorphea, Kris Warren, Bethany Jackson

**Affiliations:** ^1^School of Veterinary Medicine, Murdoch University, Perth, WA, Australia; ^2^Free the Bears, Phnom Penh, Cambodia; ^3^Diagnostic Imaging Department, Melbourne Animal Specialist Hospital, Melbourne, VIC, Australia; ^4^School of Veterinary Science, The University of Queensland, Gatton, QLD, Australia; ^5^Centre for Terrestrial Ecosystem Science and Sustainability, Harry Butler Institute, Murdoch University, Perth, WA, Australia; ^6^Centre for Biosecurity and One Health, Harry Butler Institute, Murdoch University, Perth, WA, Australia

**Keywords:** sun bear, *Helarctos malayanus*, tuberculosis, thoracic radiology, rescue center, imaging

## Abstract

Early and accurate diagnosis of pulmonary tuberculosis (TB) is key to effective outbreak management, and in humans thoracic radiography is used extensively for screening purposes. In wildlife TB radiography is a relatively accessible diagnostic tool, particularly in under-resourced settings, however its use is limited by body size. Sun bears are susceptible to human-associated TB, and their small body size makes thoracic radiography feasible. However, there are no established guidelines on normal thoracic radiographs or radiographic manifestations of TB in this species. We provide a first description of thoracic radiographs from healthy and TB affected sun bears at a bear rescue sanctuary, including correlation with postmortem results for a subset of bears. Findings of two veterinary radiologists, blinded to clinical information, revealed high agreement on broad categorization of radiographic studies as normal, abnormal, or needing correlation with further information. Agreement was lower for the presence of specific lung patterns, reflecting inherent subjectivity when classifying these features. Very few studies were identified as definitively normal, however definitively abnormal studies were significantly associated with TB cases. Diffuse bronchial and/or bronchointerstitital lung patterns were commonly reported, with a high proportion needing correlation with age and/or clinical signs to further interpret. Interstitial, interstitial-to-alveolar, alveolar and nodular lung patterns, along with radiographic signs of lymphadenomegaly and pleural fluid, were almost exclusively found in TB cases, however the sensitivity of the presence of any of these changes for detecting TB was below 70%. Radiographic reporting of thoracic lymph node enlargement detected at postmortem was low (4/17; 23%), and aortic outflow tract dilation and positional atelectasis were differential diagnoses for radiographic changes that could also represent TB. Together these findings demonstrate the importance of developing species-specific criteria for interpretation, to differentiate between common findings and manifestations of TB, and to highlight areas where radiographic techniques can be optimized to assist this. Given TB remains a global health challenge in humans and other animals (wild or domestic), and detection is key to control, we recommend development of standardized approaches to radiographic studies and their interpretation to bolster diagnostic pathways for detecting TB in sun bears, and other novel or understudied hosts.

## Introduction

Tuberculosis (TB) is an infectious airborne disease affecting humans, livestock and wildlife, with impacts on public health, livestock production, and conservation efforts. Caused by a small group of genetically similar mycobacteria belonging to the *Mycobacterium tuberculosis* complex, TB typically affects the lungs and is spread via the aerosolization of respiratory droplets (1, 2). With early diagnosis the key to managing spread (3), a major challenge to TB eradication efforts in all species is the lack of accurate and affordable screening methods to facilitate active case finding and the instigation of control measures.

Thoracic radiography is one of the oldest diagnostic approaches for human TB (4) and remains a large-scale screening tool in global control efforts, facilitated by developments in portability, computerisation, and automation (5–8). Radiographically, air-filled lungs provide good contrast for detection of typical manifestations of TB such as infiltrates, consolidation, and cavitation in the pulmonary parenchyma (9). Importantly, radiographic changes may precede clinical symptoms (10), however, despite its advantages and longevity, thoracic radiography has several limitations. There is considerable heterogeneity in the radiographic manifestations of pulmonary TB (11), and the similar appearance to other lung conditions hampers its specificity for detecting TB (12, 13). Sensitivity is sub-optimal, particularly for detecting lymph node enlargement which, in humans, can be an early indicator of TB (14–16), and cases of extra-thoracic TB will be missed entirely. Furthermore, radiographic interpretation depends on the reader, with inherent subjectivity and a lack of standardization contributing to poor inter-reader reliability in the reporting of radiographic changes when screening for human TB (12, 16, 17). In resource-poor settings, high set-up costs, inconsistent running conditions, and lack of technical expertise can create additional challenges to the generation of high-quality thoracic radiographs and their interpretation.

In veterinary practice, radiography is a ubiquitous and essential tool for the detection and characterization of thoracic disease (18). However, its use to detect TB in susceptible species is limited by patient size (19). Reports in the literature are largely restricted to the role of thoracic radiography in non-human primate models (20, 21) and in the diagnosis of sporadic TB cases in suitably sized zoo species (22–26). To enhance the use of thoracic radiography as a screening tool for TB in novel species, exploration of baseline radiological criteria and variability using thoracic radiographs from healthy and TB affected individuals is necessary to establish optimal techniques and interpretation guidelines.

Human contact was the suspected source of an outbreak of TB due to *Mycobacterium tuberculosis* in sun bears (*Helarctos malayanus*) at a bear rescue sanctuary in Cambodia, with 30 sun bears developing culture-confirmed TB between 2009 and 2019 (27). Sun bears are tropical, forest-dependent species, threatened by habitat loss and poaching, with rescue centers existing to receive bears confiscated from illegal activities (28). The median life expectancy of sun bears in captivity is 22.6 years (29). Sun bears are the smallest of the extant bear species, making thoracic radiography using portable equipment a relatively accessible tool for the investigation of thoracic disease. Further, digital images enable remote review by specialist radiologists. However, to our knowledge, there are no reports describing normal features of thoracic radiographs in sun bears.

In this study we used images from a retrospective dataset of radiographs taken during the sun bear TB outbreak to evaluate thoracic radiology as a diagnostic tool for TB in this species and setting. First, we describe the radiographic features in thoracic radiographs from sun bears with and without confirmed TB, reported by two veterinary radiologists who were blinded to patient details and clinical status. We explore agreement between the radiologists and identify a set of radiographic features found primarily in TB cases to illustrate their sensitivity and specificity for detecting TB in this population of sun bears. Second, using a subset of confirmed sun bear TB cases, we provide a descriptive correlation of radiographic features with clinical signs and post-mortem changes. Finally, we highlight key differential diagnoses for radiographic features seen in sun bears with thoracic TB. Together, the results will provide baseline data from which to build clinically relevant guidelines for the use of thoracic radiology in this and similar captive sun bear populations at risk of TB.

## Materials and methods

### Study design, setting, and population

This observational study was conducted at the Cambodia Bear Sanctuary (CBS), Cambodia (11°18'00.9''N 104°48'04.9''E). The study population comprised sun bears radiographically screened for TB at the sanctuary between February 2016 and October 2022. Sun bears were screened for TB during routine health checks and when undergoing diagnostic work ups. We used a single-center, retrospective, case-control design to investigate thoracic radiographic changes associated with TB in sun bears. A subset of radiographs from sun bears with confirmed TB were used to compare radiographic findings with clinical signs and macroscopic post-mortem changes, and to describe potential differential diagnoses when interpreting changes on thoracic radiographs.

TB cases were defined as sun bears at the CBS with bacteriologically confirmed TB based on culture of *Mycobacterium tuberculosis* from tissue/s at post-mortem examination (PME). Healthy, or “non-TB bears” were defined as sun bears without clinical signs consistent with TB or any other respiratory condition, and which remained alive and healthy at least 18 months post-radiographic study, or subsequently underwent a PME at which there were no macroscopic signs or microbiological evidence of TB. These controls were selected by the lead author (KO) from the study population if they had recent thoracic radiographs available, and to match the approximate life stage distribution of cases. A subset of TB cases and non-TB bears had more than one study available due to previous radiographic investigations, however only the most recent study for each individual bear was included when comparing radiographic features between TB cases and non-TB bears. Additionally, only TB cases with a radiographic study taken within 3 months of diagnosis were included in the comparative analyses. Treatment of bears with human anti-tuberculosis medication was not permitted and therefore had not been attempted in any cases.

### Ethics statement

This study retrospectively used radiographic and post-mortem data generated during routine veterinary and post-mortem procedures. No animals were anesthetized or euthanized for the specific purpose of this study, and all procedures were carried out using standard veterinary techniques for clinical examination, radiographic screening, and PME. Ethics approval for the use of clinical, radiographic and post-mortem data in this study was obtained from the Murdoch University Animal Ethics Committee, under permit number R3276/20.

### Radiographs

Radiographs were obtained under general anesthesia. Sun bears were immobilized in their indoor dens using a combination of zolazepam/tiletamine (1.25 mg kg^−1^) and medetomidine (0.0125 mg kg^−1^) delivered by intramuscular blow dart. Once immobilized, bears were transported to the veterinary clinic (an ~5-min journey) followed by intubation for maintenance of anesthesia with isoflurane and oxygen. Radiographs consisted of digital images, taken using a portable x-ray generator (Poskom PXP Excelray 40HF until November 2020; EcoRay HF1040 after November 2020) and an indirect computed radiography (CR) system (Agfa CR15-X; until March 2022) or a direct digital radiography (DR) system (Agfa DR 14e; after March 2022). X-ray tube settings were selected according to a sun bear thoracic radiograph protocol developed at the sanctuary (100 kVp and 3.2 mAs), with minor adjustments made if necessary, according to individual size variations. When using the CR system a table-top cassette (35 x 43 cm) was used in combination with a grid (35 x 43 cm, 40 line/cm, 12:1 ratio, focal distance range 91–106 cm). When the DR system was used, the image detector (35 x 43 cm) was placed under a Perspex table-top without an external grid. Bears were stabilized, placed in right lateral recumbency for a standard biometric photo, then in dorsal recumbency for intravenous catheter placement, followed by radiography prior to any other procedure. Radiographic studies consisted of three views (right and left lateral views, and a ventrodorsal view) and were taken by the duty veterinarian. Where possible, images were taken on maximal inspiration, with the use of positive pressure ventilation to assist lung inflation if necessary. Digital Imaging and Communications in Medicine (DICOM) format files were available for all radiographic studies, apart from one study which had JPEG files only.

### Radiographic interpretation

Each radiographic study was assigned a computer-generated number to randomize the viewing order (30), and two board-certified veterinary radiologists (NW and AR) were given access to the de-identified electronic files. The radiologists were blinded to bear signalment and clinical status, to the timing of radiographic studies, and to each other's interpretation. Radiologists provided comments for each study under the following anatomic region headings: skeletal, cardiovascular structures, lungs, pleural space, mediastinum, other. Additionally, each radiologist indicated if they considered the study to be abnormal and clearly consistent with disease. The term “disease” was used rather than “TB” specifically, given the lack of published criteria for the radiographic appearance of TB in sun bears.

Radiologist findings were coded by the lead author (KO). If the study was highlighted as abnormal and clearly consistent with disease, it was coded as “abnormal.” If the study was reported as within normal limits (WNL), or the only change reported was clearly stated as due to positional atelectasis, the study was coded as “WNL.” The remaining radiographs were coded as “correlation needed”, indicating that further information (such as age and/or clinical signs) was needed to clarify if the changes identified were normal or abnormal. Radiographic lung patterns were coded as “reported” or “not reported,” except for bronchial and bronchointerstitial patterns which were coded as “not reported,” “mild,” “moderate,” or “marked,” based on the use of these terms by the radiologist. Lymph node enlargement and pleural fluid were coded as “reported” or “not reported,” with “reported” studies including those where lymph node enlargement/pleural fluid was considered possible due to signs consistent with their presence being detected. If a lung pattern was clearly stated by the radiologist to be due to atelectasis, atelectasis was coded as “reported” instead of the corresponding lung pattern. However, if atelectasis was queried as only one of several possible causes for a stated lung pattern, the lung pattern was coded as “reported.”

### Clinical signs and postmortem findings

For all bears, recorded clinical history, husbandry observations, and monthly body weights were reviewed, and for TB cases post-mortem reports and photographs were reviewed. A set of criteria for relevant clinical signs and post-mortem findings were developed (Supplementary Table 1) to allow grading of clinical signs, lung post-mortem findings, lymph node post-mortem findings, and pleural fluid post-mortem findings as mild (+), moderate (++), marked (+++), when present.

### Statistical analyses

Distributions and demographic data for TB cases and non-TB bears were explored using the Shapiro-Wilk test for normality (age, body condition score), and significance of differences between the groups determined by Kruskal-Wallis (age, body condition score) or Yates corrected chi-square (sex). The significance of differences between the outcome of TB cases or non-TB bears, and radiographic findings of WNL, needing correlation, or abnormal, were determined by Fisher's exact test (2-tailed; any category < 5 studies) or by Yates corrected chi-square (all categories >5 studies). Percentage agreement (PA) between radiologist findings was calculated as a raw percentage using the formula:


$$\begin{array}{l} PA=\frac{\left|value\;1-value\;2\right|}{\left(\left(value\;1+value\;2\right)/2\right)}\;*\;100 \end{array}$$

and further explored using Cohen's kappa (K), which was calculated according to:


$$\begin{array}{l} K=\frac{\left(Po-Pe\right)}{\left(1-Pe\right)} \end{array}$$

where P_o_ was the relative observed agreement between radiologists, and P_e_ was the hypothetical probability of chance agreement. Cohen's kappa was interpreted according to the following levels of agreement (31): 0–0.20 = negligible; 0.21 – 0.39 = minimal; 0.40 – 0.59 = weak; 0.60 – 0.79 = moderate; 0.80 – 0.90 = strong; >0.90 = almost perfect. Correlations between select radiographic findings and TB cases or non-TB bears were calculated using raw proportions and odds ratios with 95% CI. Radiographic findings associated primarily with TB cases were then used to calculate the sensitivity and specificity of such findings, using Epitools, Ausvet, Australia (32).

## Results

### Study population

**Figure 1:**
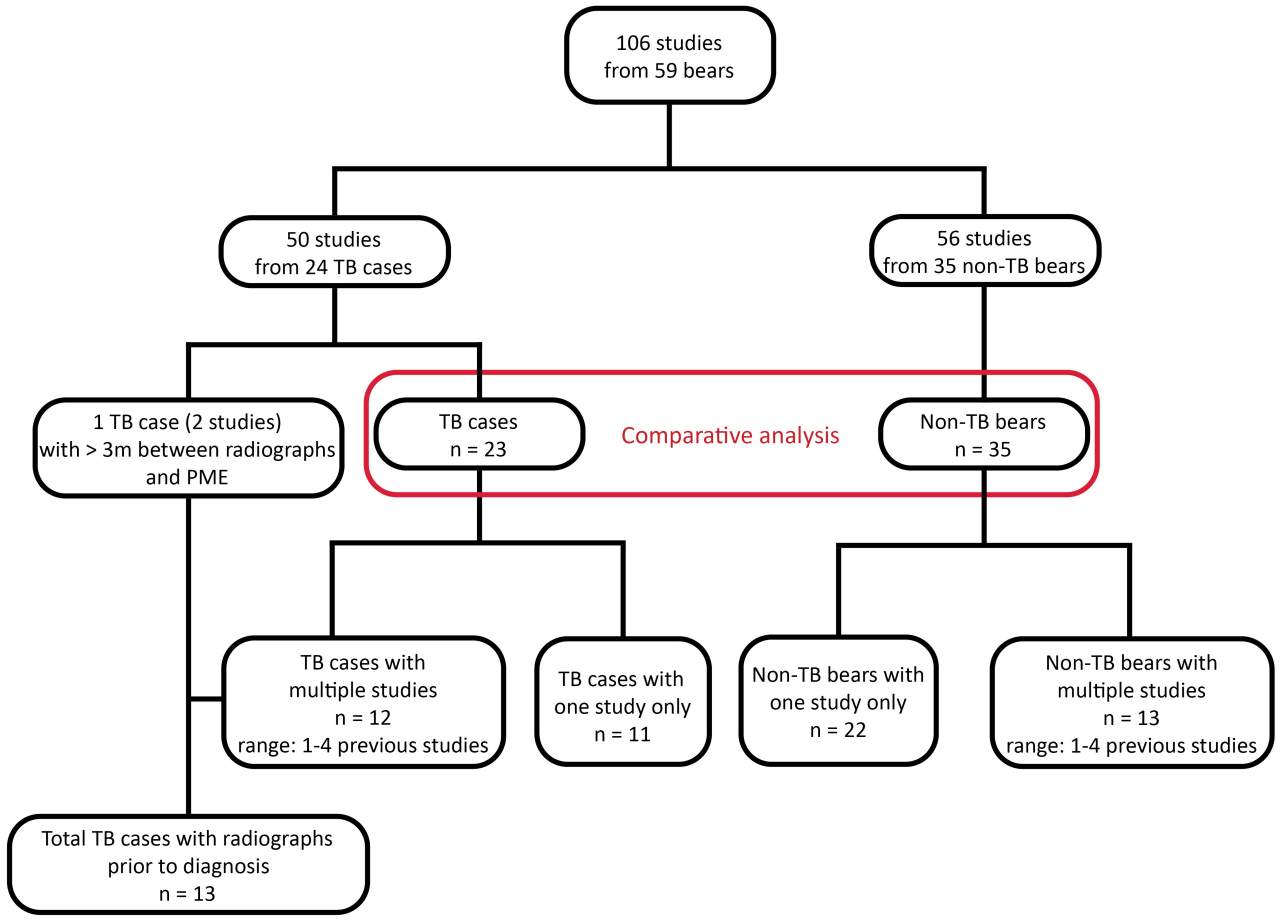
Flow chart showing study population and available radiographic studies from sun bears (*Helarctos malayanus*) at the Cambodia Bear Sanctuary. TB, tuberculosis.

A total of 106 radiographic studies representing 59 bears were reviewed, including 50 studies from 24 TB cases and 56 studies from 35 non-TB bears (Figure 1). Multiple studies taken at different time points were available for 13 TB cases and 13 non-TB bears. There were no significant differences between the age and sex of TB cases and non-TB bears, however TB cases had significantly lower body condition scores (Kruskal-Wallis test, *p* = 0.01) compared with non-TB bears (Table 1).

**Table 1:** Comparison of age, sex, and body condition between sun bears (*Helarctos malayanus*) with tuberculosis (TB cases) and sun bears with no indications of tuberculosis (non-TB bears).

	**TB cases (*n* = 23)**	**Non-TB bears (*n* = 35)**	***p*-value**
Age (years)	14.5 ± 7.8^*^	12.8 ± 8.1^*^	*p* = 0.46^#^
BCS (out of 5)	2.4 ± 0.7^*^	2.8 ± 0.5^*^	*p* = 0.01^#^
Sex (male:female)	10:13	9:26	*p* = 0.26^∧^

BCS, body condition score.

^*^Data are presented as mean ± standard deviation.

^#^Kruskal-Wallis test.

^∧^Yate's corrected Chi-squared.

### Radiographic findings across all studies

#### Normal vs. abnormal

For the full dataset of 106 radiographic studies including multiple studies for 26 bears, few studies were reported as WNL by either radiologist [radiologist 1 (R1): 11% (12/106), radiologist 2 (R2): 2% (2/106)]. The proportion of radiographs with changes that needed correlation with further information ranged from 67% (71/106, R1) to 86% (91/106, R2), while those reported as definitively abnormal ranged from 12% (13/106, R2) to 22% (23/106, R1).

#### Radiographic features

Across the full dataset, a bronchial or bronchointerstitial lung pattern was reported by one or both radiologists in 97% (103/106) of studies, being described as mild or moderate in 95% (98/103) and marked in 5% (5/103) of these studies. Any of interstitial, interstitial-to-alveolar, alveolar, or nodular patterns were reported in 21% (22/106) of studies, and signs consistent with lymph node enlargement in 5% (5/106) of studies. Atelectasis was reported by one or both radiologists in 54% (57/106) of thoracic studies, with the majority of these (65%, 37/57) reported to be right-sided. The only cardiovascular structural finding was aortic outflow tract enlargement, which was mentioned as present or possibly present by one or both radiologist in 65 studies from 39 bears.

### Radiographic findings in TB cases and non-TB bears

#### Normal vs. abnormal

Considering the most recent radiographic study and TB-status for each bear, radiologists reported definitively abnormal radiographs in a similar proportion of TB cases (R1: 61%, R2: 52%), with strong agreement (K = 0.82), and an abnormal finding was significantly associated with TB positive bears (*p* < 0.0001, Table 2). Comparatively few non-TB bears were reported to have definitively abnormal radiographs (R1: 17%, R2: 0%), with the majority of studies from non-TB bears reported as needing correlation with additional information (R1: 68%, R2: 100%). Radiologists reported similar proportions of radiographs from TB cases needing correlation (R1: 35%, R2: 47%), with moderate agreement (K = 0.74). The need to correlate radiographic findings with further information was not significantly associated with TB-status. Only one study from a TB case was considered WNL by R1, with the same radiologist considering 14% (5/35) of studies from non-TB bears definitively WNL. R2 did not report any studies to be definitively WNL from either group (0/58).

**Table 2:** Categorization of sun bear (*Helarctos malayanus*) thoracic radiograph studies by two radiologists, including agreement between radiologists and comparison between categorization level and tuberculosis (TB) status.

**Category**	**Studies from TB cases** ***n*** = **23**			**Studies from non-TB bears** ***n*** = **35**		**Comparison Odds ratio (95% CI)** ***p*****-value**	
	**R1**	**R2**	**K**	**R1**	**R2**	**R1**	**R2**
WNL	1	0	n/a	5	0	n/a	n/a
Correlation needed	8	11	0.74	24	35	vs. *WNL*	vs. *WNL*
						1.67 (0.17, 16.5)	0.33 (0.02, 5.75)
						*p* = 1^#^	*p* = 0.46^#^
Abnormal	14	12	0.82	6	0	vs. *WNL*	vs. *WNL*
						10.8 (1.03, 114.15)	13.0 (0.42, 405)
						*p* = 0.06^#^	*p* = 0.24^#^
						vs. *WNL/correlation*	vs. *WNL/correlation*
						7.52 (2.23, 25.3)	39.0 (4.61, 330)
						*p* < 0.0001^*^	*p* < 0.0001^#^

K, Cohen's kappa; WNL, within normal limits; R1, radiologist 1; R2, radiologist 2.

^#^Fisher's exact test (2-tailed).

^*^Yates corrected Chi-square.

#### Radiographic features

Table 3 summarizes radiographic features reported by both radiologists in the most recent study from each bear. Both radiologists reported a bronchial lung pattern in 40% (23/58) of studies, and a bronchointerstitial pattern in 38% (22/58, R1) to 46% (27/58, R2) of studies, with neither finding significantly associated with TB status. Agreement between radiologists reporting a bronchial lung pattern ranged from negligible (TB cases: K = 0.04) to minimal (non-TB bears: K = 0.30). Similarly, agreement when reporting a bronchointerstitial lung pattern ranged from negligible (TB cases, K = 0.11) to minimal (non-TB bears, K = 0.22). When the severity of bronchial and bronchointerstitial lung patterns was examined, there were five studies with either of these patterns reported as marked, and all were from TB cases.

**Table 3:** Agreement between two radiologists reporting select features in radiographs from sun bears (*Helarctos malayanus*) with TB (TB cases) and sun bears with no indications of TB (non-TB bears).

	**Studies from TB cases** ***n*** = **23**			**Studies from non-TB bears** ***n*** = **35**		
	**R1**	**R2**	**% agreement K**	**R1**	**R2**	**% agreement K**
**Lung pattern**						
Bronchial	8	8	56.5	15	15	65.7
			0.04			0.30
Bronchointerstitial	8	7	60.9	14	20	60.0
			0.11			0.22
Interstitial	2	4	82.6	0	1	97.1
			0.24			n/a
Interstitial-to-alveolar	3	4	87.0	0	0	100
			0.50			n/a
Alveolar	8	4	65.2	0	0	100
			0.13			n/a
Nodular	2	3	95.6	0	0	100
			0.76			n/a
**Lymph nodes**						
Signs consistent with lymph node enlargement reported	3	4	95.6	0	0	100
			0.83			n/a
**Pleural fluid**						
Signs consistent with pleural fluid reported	6	0	0	0	0	100
						n/a
**Atelectasis**						
Signs consistent with atelectasis reported	10	9	78.3	11	15	88.6
			0.55			0.76

K, Cohen's kappa.

### Accuracy of select radiographic features

The following radiographic features were almost exclusively associated with TB cases: interstitial, interstitial-to-alveolar, alveolar, and nodular lung patterns; signs consistent with lymph node enlargement; and signs consistent with pleural fluid. The presence of one or more of these radiographic features was highly specific for detecting TB, however lacked sensitivity (Table 4). The sensitivity improved slightly if the opinions of both radiologists were considered together, in parallel.

**Table 4:** Sensitivity and specificity of select radiographic features for diagnosing tuberculosis (TB) in sun bears (*Helarctos malayanus*) when reported by one or both radiologists.

	**Studies from TB cases *n* = 23**	**Studies from non-TB bears *n* = 35**	**Sensitivity % (95% CI)**	**Specificity % (95% CI)**
Features reported (R1)	15	0	65.2	100
			(42.7, 83.6)	(90, 100)
Features reported (R2)	14	1	60.9	97.1
			(38.5, 80.3)	(85.1, 99.9)
Features reported in parallel (R1 or R2)	16	1	69.6	97.1
			(47.1, 86.8)	(85.1, 99.9)
Features reported in series (R1 and R2)	12	0	52.2	100
			(30.6, 73.2)	(90, 100)

R1, radiologist 1; R2, radiologist 2; features = any of interstitial, interstitial-to-alveolar, alveolar, or nodular lung patterns, signs consistent with lymph node enlargement, signs consistent with pleural fluid.

### Correlation between radiographic features, clinical signs, and postmortem findings

#### Clinical signs

Eighteen of the 23 TB cases were displaying clinical signs at the time of diagnosis (Supplementary Table 2), including five with mild (+), four with moderate (++) and nine with marked (+++) signs, along with five cases with no clinical signs reported. Of the nine TB cases with marked (+++) clinical signs, all were reported to have definitively abnormal thoracic radiographs by one (*n* = 1) or both (*n* = 8) radiologists, and all had moderate (++) or marked (+++) lung changes reported at PME. Of the 10 TB cases with mild (+) or no clinical signs, three had abnormal radiographs reported. Of these three cases, one had marked lymph node enlargement which was detected radiographically (SB027), and another had a nodular lung pattern (SB058). The remaining case (SB088) had interstitial-to-alveolar and alveolar lung patterns reported, and pleural fluid was also detected.

#### Postmortem findings

A total of 83% (19/23) of TB cases had lung changes seen at PME, and 68% (13/19) of these had abnormal thoracic radiographs reported by either or both radiologists (Supplementary Table 2). Of the six cases with lung changes at PME that did not have abnormal radiographs reported, one had marked (+++) PME changes. When considering the radiographic lung patterns seen exclusively in TB cases (interstitial-to-alveolar, alveolar, nodular), the 14 TB cases reported to have one or more of these patterns all had moderate (++) or severe (+++) lung changes on PME. None of these radiographic lung patterns were seen in the six TB cases with mild (+) or no gross changes in the lung seen on PME. Four bears had normal lungs at PME, and of these, three had thoracic lymph node enlargement and the remaining bear (SB061) had no thoracic pathology reported, with *M. tuberculosis* cultured from non-healing wound tissue and lymph nodes only.

#### Lymph node enlargement and pleural effusion

Radiographic changes consistent with lymph node enlargement were identified in four of the 17 TB cases with enlarged lymph nodes described on PME, with each of these four cases having marked enlargement (+++) of tracheobronchial lymph nodes (Supplementary Table 2). One of the three cases with lymph node enlargement in the absence of any lung changes at PME was flagged radiographically (SB027). Six of the 11 TB cases noted to have an increased volume of pleural fluid at PME were queried to have scant pleural fluid visible radiographically.

### Radiographic series in TB cases

**Figure 2:**
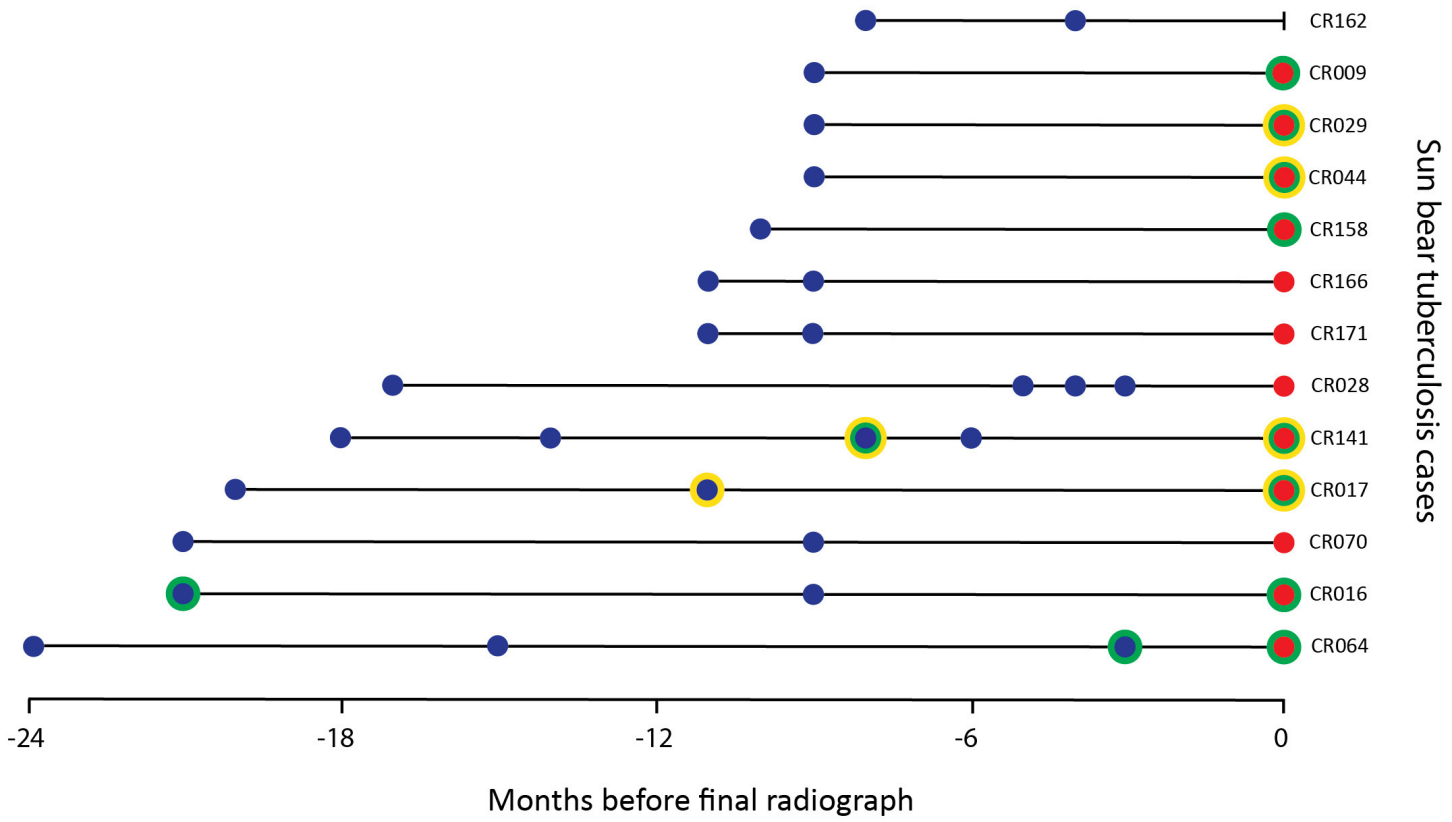
Timing of radiographic studies from 13 sun bears (*Helarctos malayanus*) with tuberculosis including those categorized as abnormal (green circle) and/or with lymph node changes (yellow circle) by one or both radiologists. A blue dot indicates studies taken before final diagnosis by culture, a red dot indicates study taken at diagnosis by culture.

Additional thoracic radiographic studies, from 3 to 24 months prior to diagnosis, were available for 13 TB cases (Figure 2). Three of these prior studies were reported as definitively abnormal by a single radiologist, including one from 3 months prior to diagnosis. The other two were taken 8 and 20 months before diagnosis, with both cases having a subsequent study considered WNL or needing correlation. Two of the four TB cases with enlarged lymph nodes detected in their final study had enlarged lymph nodes questioned in a previous study.

### Radiographic differential diagnoses

#### Aortic outflow tract dilation

**Figure 3:**
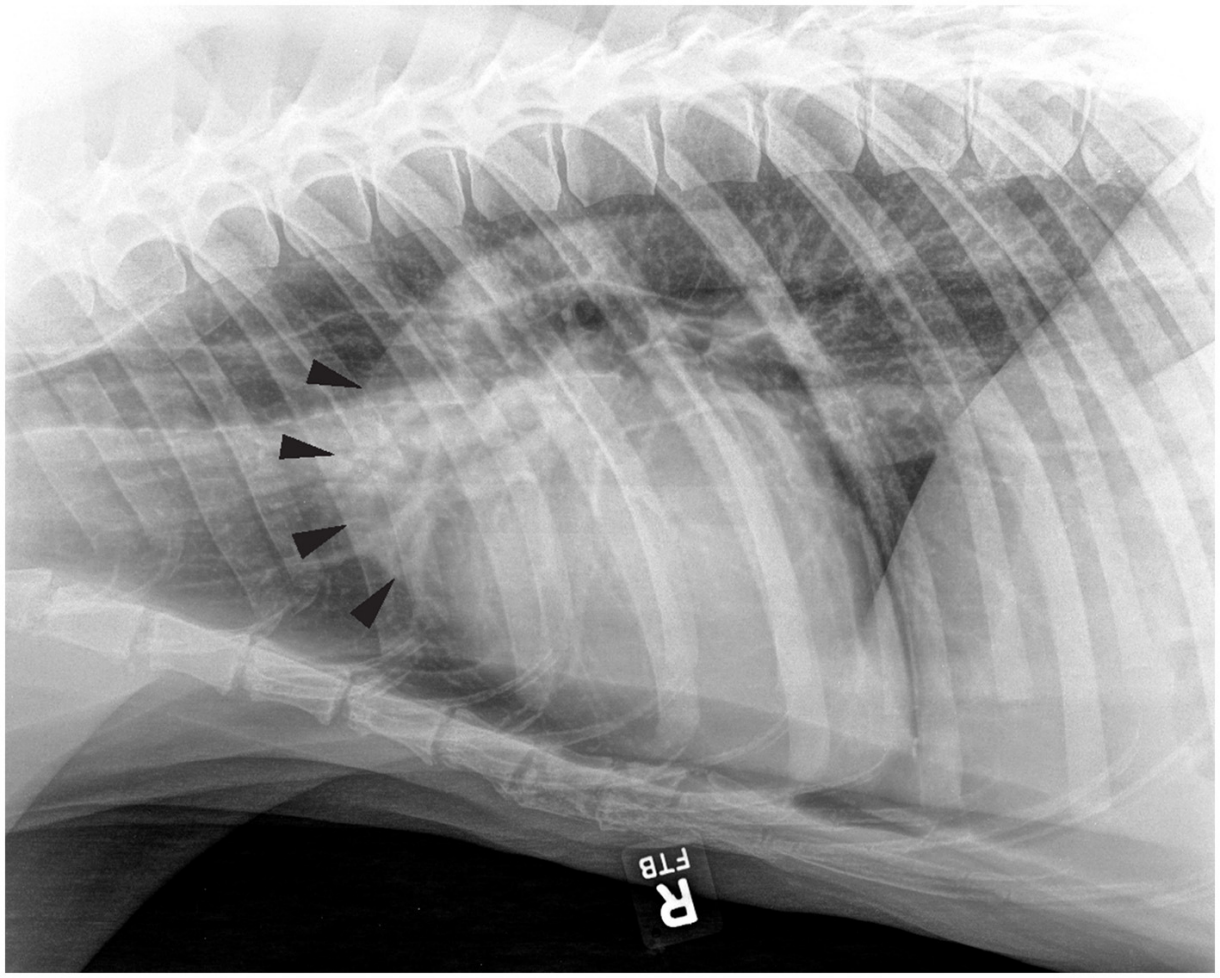
Right lateral thoracic radiograph from a sun bear (*Helarctos malayanus*) without tuberculosis (CR021) showing evidence of aortic outflow tract dilation (arrows).

**Figure 4:**
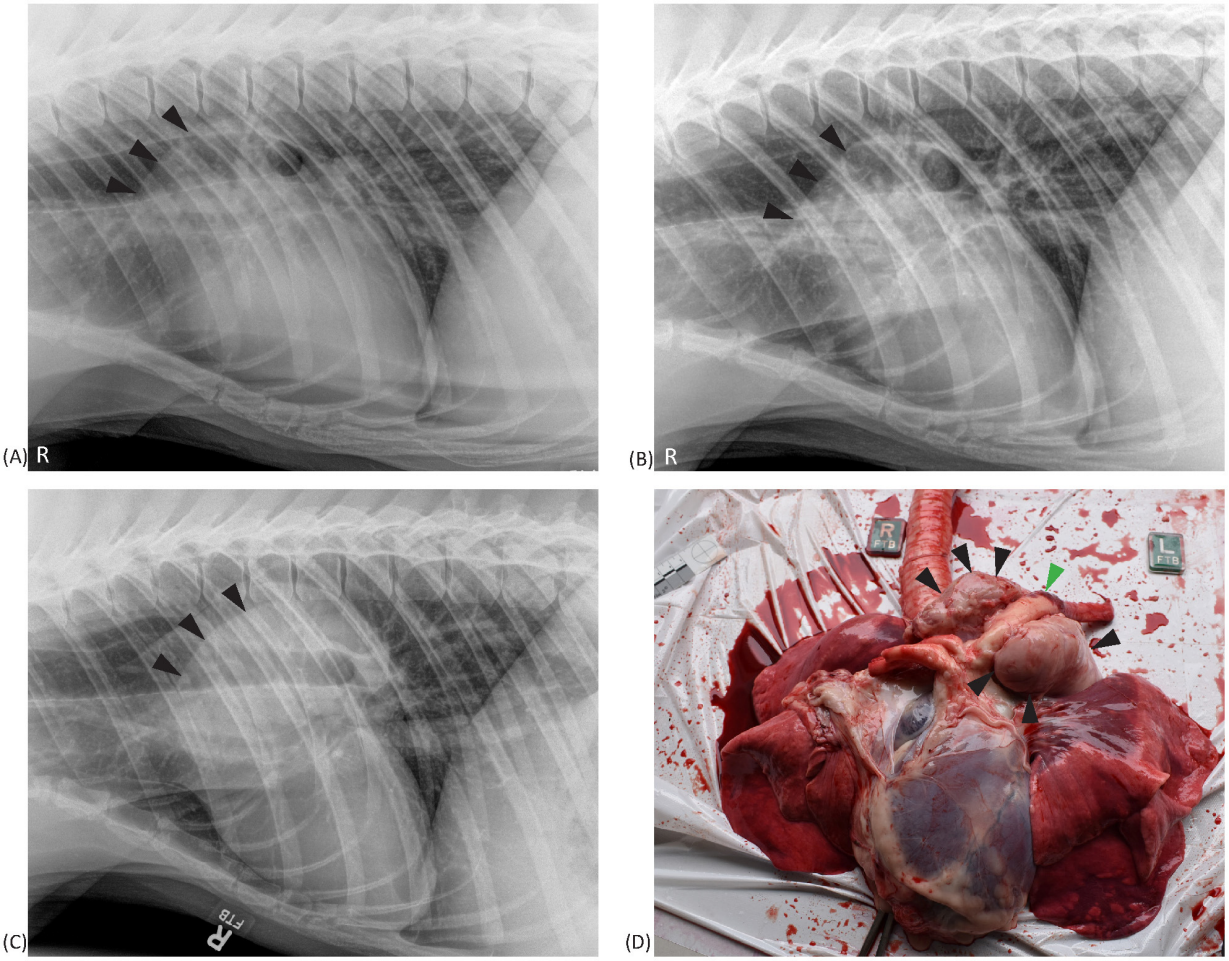
Right lateral thoracic radiographs and a postmortem image from a sun bear (*Helarctos malayanus*) tuberculosis case (CR141). Radiographs show increased cranial hilar soft tissue opacity (black arrows) at 8 months prior **(A)** and 6 months prior **(B)** to postmortem, and on the day of postmortem **(C)**. The postmortem photograph **(D)** shows marked tracheobronchial lymph node enlargement (black arrows) surrounding the aorta (green arrow).

**Figure 5:**
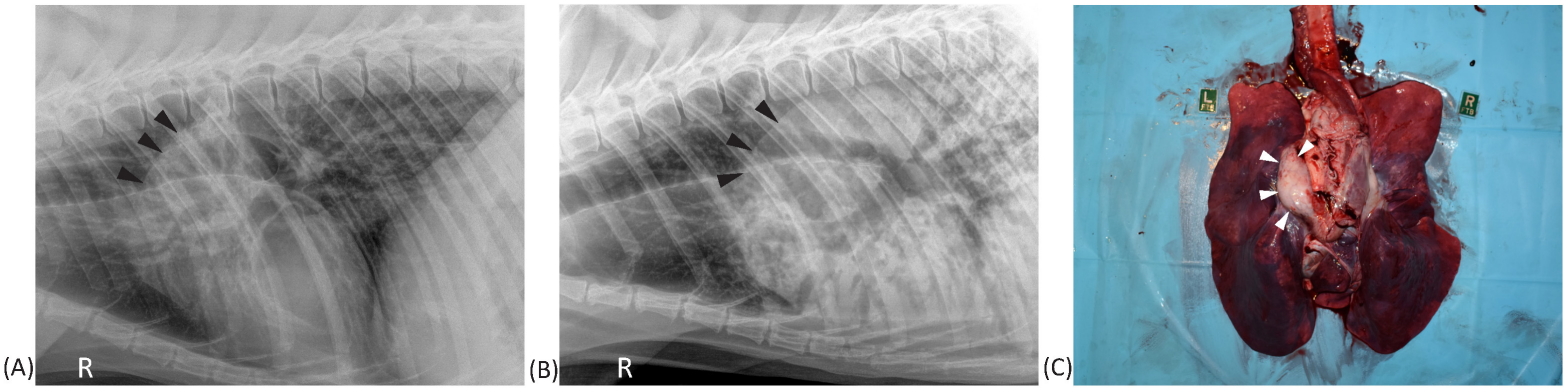
Right lateral thoracic radiographs and postmortem image from a sun bear (*Helarctos malayanus*) tuberculosis case (CR017). **(A)** Radiograph taken 11 months prior to postmortem showing increased soft tissue opacity craniodorsal to the heart base (black arrows). **(B)** Radiograph taken on the day of postmortem showing advancement of the same soft tissue opacity along with marked pulmonary changes. **(C)** Postmortem photograph showing marked tracheobronchial lymph node enlargement (white arrows).

Aortic outflow tract dilation and lymph node enlargement were mentioned as co-occurring differential diagnoses by both radiologists in four studies from three bears. Aortic outflow tract dilation was reported by both radiologists in healthy non-TB bears (for example see Figure 3). Serial radiographs (8 months prior, 6 months prior, and at the time of PME) from a single TB case with marked tracheobronchial lymph node enlargement are shown in Figure 4. Both radiologists were suspicious of lymph node enlargement in the earliest radiograph (4A) and both prioritized this diagnosis on the final radiograph (4C) while mentioning atypical (R1) and severe (R2) aortic dilation as a possible differential. On radiograph 4B, lymphadenopathy was not mentioned by either radiologist, although mild aortic dilation was considered to be present. Figure 5 provides a second series of radiographs, with aortic dilation prioritized by both radiologists in 5A, 11 months prior to PME, with atypical lymph node enlargement a differential diagnosis. In 5B, the cardiac silhouette was effaced by severe pulmonary changes making it impossible to differentiate between lymph node enlargement and aortic dilation to explain the hilar soft tissue opacity, with PME confirming lymph node enlargement (5C).

#### Positional atelectasis

**Figure 6:**
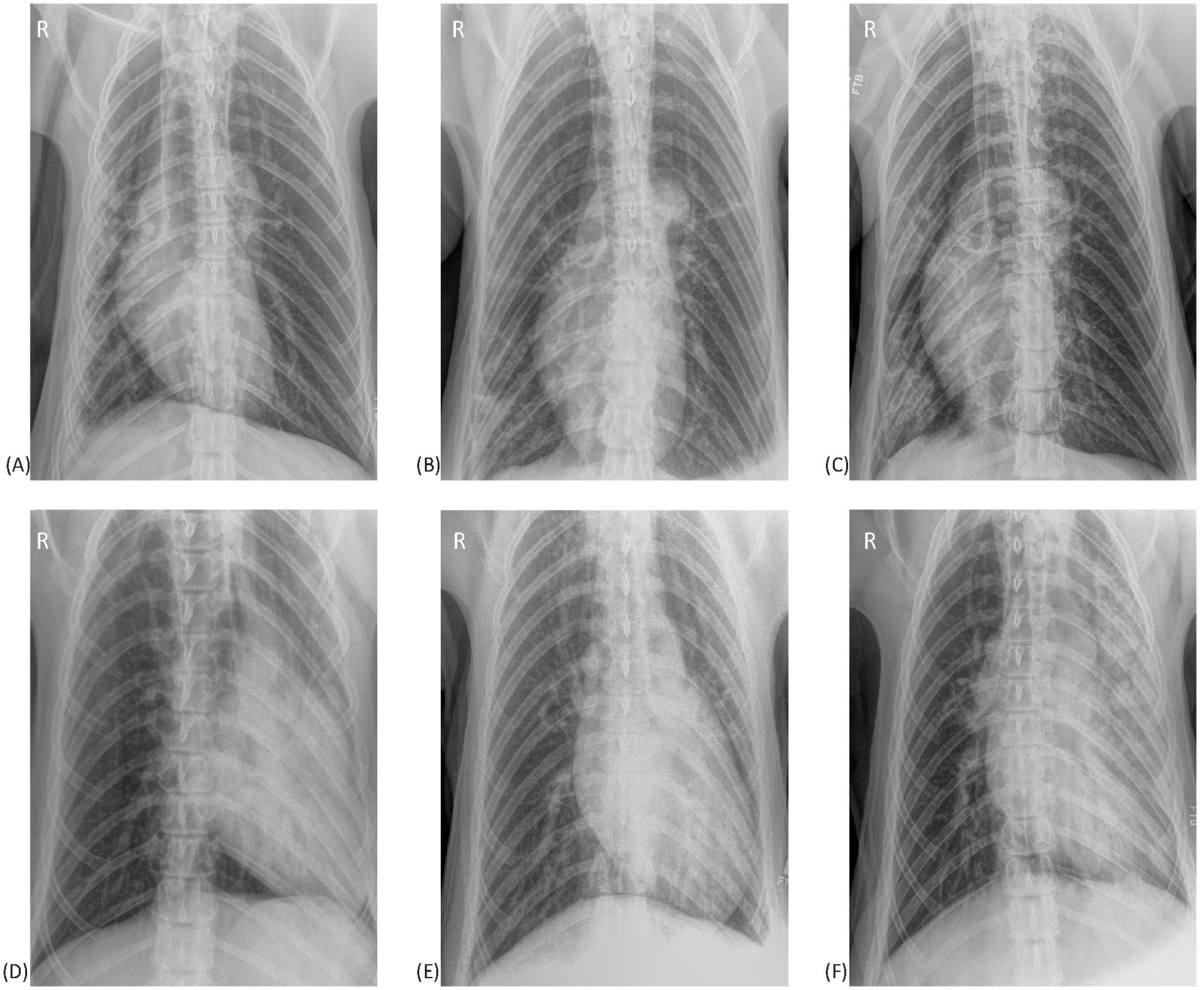
Ventrodorsal thoracic radiographs from six sun bears (*Helarctos malayanus*). **(A)** Radiograph from a sun bear without tuberculosis showing right sided atelectasis causing patchy interstitial changes in the right lung and a mediastinal shift to the right. **(B)** Radiograph from a sun bear tuberculosis case showing patchy interstitial-to-alveolar opacities in the caudal right lung. Note the lack of a mediastinal shift. **(C)** Radiograph from a sun bear tuberculosis case with increased opacity in the right lung, reduced right lung volume and mediastinal shift to the right. **(D)** Radiograph from a sun bear without tuberculosis showing marked left-sided atelectasis. Note the severely reduced left lung volume and leftward mediastinal shift. **(E)** Radiograph from a sun bear tuberculosis case showing increased opacity in the left lung, reduced left lung volume and mediastinal shift to the left. **(F)** Radiograph from a sun bear tuberculosis case showing marked patchy interstitial-to-alveolar pattern in the left lung, reduced left lung volume, and leftward mediastinal shift.

Figure 6 illustrates examples of overlapping pulmonary changes that may be present in both TB cases and non-TB bears. Examples of positional atelectasis causing pulmonary changes on radiographs from non-TB bears are shown in 6A and 6D. Similar changes are seen in TB cases in the remaining images, all of which had moderate to severe lung pathology on PME within 3 months of the radiograph. One of the studies from a TB case (6B) was considered abnormal and indicative of disease by a radiologist, with another (6F) reported to have atelectasis that could be masking concurrent true pathology. In the remaining images from TB cases the pulmonary changes were considered consistent with positional atelectasis by radiologists.

## Discussion

Using thoracic radiographs obtained during a TB outbreak at a rescue center in Cambodia, we investigated the radiographic features of TB in sun bears. Thoracic radiographs considered definitively abnormal by two blinded radiologists were significantly correlated with diagnosed TB cases. Certain radiographic lung patterns were primarily reported in TB cases and were associated with indicators of advanced disease such as increased severity of clinical signs and thoracic pathology on PME. Along with lymph node enlargement and pleural fluid accumulation, these radiographic features were highly specific for TB in sun bears, however their sub optimal sensitivity cautions against ruling TB out in their absence. We use the results of this study to draw attention to common findings in healthy sun bear chests, such as mild, diffuse, bronchial and/or bronchointerstitial lung patterns, and to highlight interpretive challenges posed by differential diagnoses for select thoracic findings, such as aortic dilation and positional atelectasis. Given increasing reports of the spill-over of human TB at human-wildlife interfaces such as rescue centers and zoos (19, 33–36) alongside the established challenges of antemortem TB diagnosis, we illustrate the importance of leveraging screening radiographs from healthy and diseased individuals to develop baseline normal and diagnostic criteria that optimize management of TB in novel, often understudied, hosts.

Almost all studies had a radiographic lung pattern reported, however certain patterns were ubiquitous (bronchial, bronchointerstitial) while those reflecting consolidation, infiltration, and granulomatous pulmonary disease (e.g., interstitial, interstitial-to-alveolar, alveolar, nodular) were found almost exclusively in TB cases. Given these lung changes are typical manifestations of TB (37–41), this result was not surprising, nor was the association between these lung patterns and cases with moderate to severe macroscopic changes at PME. Thoracic lymphadenopathy and pleural effusion are also common manifestations of TB (42–44) and were likewise predictably reported only in TB cases. Agreement between the two radiologists when reporting the TB-associated lung patterns ranged from negligible (K = 0.13, alveolar) to moderate (K = 0.76, nodular). The specialist-level qualification of both radiologists makes it less likely that this lack of agreement was experience-related, although one radiologist did have prior experience reviewing radiographs from bear species. However, the known inherent subjectivity in reporting radiographic lung patterns (45–47) plausibly contributed, with agreement rising to strong (K = 0.82) when determining if changes represented a definitive departure from normal. Higher agreement is reported between radiologists evaluating human thoracic radiographs for TB when asked to determine the presence or absence of clinically important changes, compared with the presence or absence of specific subcategories of abnormalities (48, 49). From a clinical perspective in the context of screening sun bears for TB, reliable identification of radiographs from diseased animals is of clear practical relevance, regardless of how a specific lung pattern is reported. This has implications for TB management in other novel species and under-resourced settings, suggesting that a focus on determining clear radiographic indicators of disease, and optimizing radiographic techniques to identify them, may enhance thoracic radiography as a screening tool.

In contrast to the TB-associated lung patterns, bronchial and bronchointerstitial patterns were commonly reported in radiographs from bears with and without TB, although we note all studies with these patterns graded as “marked” were from TB cases. This highlights the potential value in developing standardized grading of such patterns, given increasing severity is more likely to be associated with disease, whilst the significance of mild or moderate changes should be cautiously interpreted. Mild or moderate bronchial or brochointerstitial lung patterns in the absence of any other changes accounted for a high proportion of the radiographs reported as needing correlation with further information, with very low agreement on which studies showed a specific pattern, again underscoring subjectivity in lung pattern assignment. Agreement studies in thoracic radiology consistently show lower agreement between radiologists when determining the absence, rather than presence, of significant changes, reflecting uncertainty arising from a spectrum of possible variations of normal (50–52). Given that bronchial and bronchointerstitial patterns are non-specific and can be a normal finding, particularly in older animals (53), the blinding of the radiologists to demographic and clinical information in this study likely underlies a hesitation to report radiographs with these lung patterns as WNL. This is supported by both veterinary and human studies that show radiologist knowledge of clinical and patient background can impact the interpretation of thoracic radiographs (54, 55). It is also possible that radiographic technique contributed to an overreporting of these patterns, including the relative size of sun bears leading to underexposure of radiographs and lung parenchymal patterns appearing artifactually more bronchial/bronchointerstitial (56, 57).

While thoracic radiography clearly demonstrated features that were highly specific for TB, several shortcomings were observed, including suboptimal sensitivity, low detection of lymph node changes, and questionable accuracy in detecting early changes in TB cases. Although abnormal radiographs were flagged from every TB case displaying marked clinical signs, thoracic radiographs from over a third (9/23) of TB cases, particularly those with mild or no clinical signs, were not considered definitively abnormal by either radiologist. Given detection of TB prior to obvious illness is key to optimizing treatment and control measures, the low radiological detection of sun bear TB cases with mild or no clinical signs contributes to suboptimal sensitivity in this situation, as for human TB (58, 59). We further explored radiographic detection of early disease by evaluating TB cases with radiographic studies prior to postmortem confirmation of disease. Only three had previous radiographs considered abnormal, and an additional one had enlarged lymph nodes queried. Given infection with *M. tuberculosis* often follows a chronic course (60) it is expected that a proportion of the cases were already infected, and may have had disease, when one or more previous radiographs were taken. The apparent challenge and inconsistency in early radiographic detection suggests that increased frequency of radiographic screening may improve detection of changes when they first manifest and allow immediate interventions to minimize risk of transmission to other bears. However, the requirement for general anesthesia puts practical constraints on screening frequency, particularly in facilities with high numbers of at-risk bears. Given TB in sun bears can manifest as purely extra-thoracic disease (e.g., SB061 with cutaneous and abdominal disease only), as shown with *Mycobacterium bovis* infection in other species (61–64), the sensitivity of thoracic radiology as an isolated diagnostic tool in this specific scenario is implicitly poor and supports the use of a multi-modal approach to TB diagnosis in all species. Finally, caution is warranted when extrapolating the demonstrated high specificity of the select radiographic features to other populations where the risk of non-TB pulmonary conditions may differ to this study setting.

Although thoracic lymph node enlargement was noted at PME in over 70% of TB cases, radiographic detection only occurred with marked enlargement of the tracheobronchial nodes and had the additional challenge of being radiographically similar to aortic dilation. Lymph node enlargement is an early predictor of TB in other species (65–68) and can manifest before infection has progressed in the lung to become transmissible, underscoring the advantage of early and accurate detection of lymphadenopathy. Sun bear thorax size is at the upper limit of what effectively fits on a large sized X-ray cassette/detector, so it is possible that suboptimal imaging of the cranial thorax affected the ability to radiographically detect cranial mediastinal lymphadenomegaly. Guidelines to ensure bear positioning and radiographic technique are optimized for visualization of relevant lymph nodes are indicated. Aortic dilation as a differential diagnosis may reflect radiologist bias stemming from reports of cardiovascular disease as a leading cause of death in rescued bears in Asia (69), and radiographic accuracy may benefit from the development of anatomical and radiological guides to differentiate increased soft tissue opacity in the hilar region (70). Use of cross-sectional imaging modalities such as ultrasound and/or ideally computed tomography, would enhance the ability to detect subtle lymph node changes (16, 68, 71). While access, financial capacity and expertise/training are barriers to adopting these approaches in resource-poor settings, their use in the development of radiographic guidelines and appropriate anatomical references for this species could be considered.

Atelectasis was a common finding in radiographs from both TB cases and non-TB bears, likely resulting from dependent lung collapse exacerbated by positioning and general anesthesia (18). While the relatively large body mass of sun bears, the necessity for general anesthesia, and time in lateral recumbency during immobilization and transport are all unavoidable contributors to positional atelectasis, its presence complicates radiographic interpretation. Examples from this study (Figure 6) illustrate how the resulting increase in lung opacity could be mistaken for disease, or conversely, true pathologic changes may be falsely attributed to atelectasis or obscured by border effacement if occurring concurrently. Guidelines for sun bear thoracic radiography should include measures to minimize the impact of atelectasis, including technique (prioritizing radiography as early as possible after immobilization, the use of positive pressure ventilation to ensure inspiratory images, repetition of ventro-dorsal view after lateral views) and interpretation criteria (such as presence/absence of a mediastinal shift/cranial displacement of diaphragmatic crus, and presence/absence of air bronchograms) to differentiate anesthesia-induced atelectasis from consolidation caused by disease processes.

Limitations of this study include the low number of radiographic studies, and inconsistencies in technical aspects due to equipment changes and a team of clinicians rather than the same individual performing radiographic acquisition. While the low numbers mean we are unable to make robust generalizations of our findings beyond this population, our sample size was determined by the available radiographs and number of TB cases. The technical inconsistencies were inherent in the retrospective study design and reflect the realities of capturing clinical, non-experimental data in a rescue center context.

## Conclusions

This study contributes to knowledge of common and potentially normal findings on sun bear thoracic radiographs and interpretation challenges for non-specific abnormal findings, concluding that the sensitivity of thoracic radiography is sub optimal for early detection of TB in this species. Accessible species-specific baseline information can be scarce for species such as sun bears that are relatively rare globally, highlighting the importance of generating this information from, and ultimately for the benefit of, the sizeable captive populations in high TB burden regions. We define a set of radiographic lung patterns and other thoracic features associated with TB cases, and recommend the definition and adoption of techniques that minimize positional atelectasis, optimize exposure factors, ensure inclusion of the entire thorax on all views, and facilitate visualization and identification of enlarged lymph nodes, to enhance thoracic radiography as a relatively accessible screening tool. Accessibility and accuracy are strengthened by the relative ease with which multiple specialist opinions can be sought digitally. We propose that our findings are incorporated into the development of guidelines for standardized radiographic techniques and interpretation criteria to bolster diagnostic pathways for detecting TB in sun bears, and potentially other novel and understudied wildlife hosts of TB.

## Data Availability

The raw data supporting the conclusions of this article will be made available by the authors, without undue reservation.
