# Supplementary material for: Comparative thoracic radiography in healthy and tuberculosis-positive sun bears (Helarctos malayanus)
Source: Front Vet Sci. 2025 Jan 6;11:1460140. doi: 10.3389/fvets.2024.1460140 (PMC11743561; doi:10.3389/fvets.2024.1460140)
Supplement: Supplementary file 1 [file Table_1.pdf]

Supplementary Table 1. Parameters used to grade clinical signs and postmortem findings in tuberculosis positive sun bears (*Helarctos malayanus*) at a sanctuary in Cambodia.

Criteria	Grade		
	Mild +	Moderate ++	Marked +++
**Clinical signs**<br>**Weight loss**<br>*+ up to 5kg loss in past 3 months*<br>*++ 5 – 10kg loss in past 3 months*<br>*+++ >10kg loss in past 3 months*<br>**Appetite reduction**<br>*+ reduction reported in past 1 week*<br>*++ reduction reported in past 1-4 weeks*<br>*+++ reduction reported for > 4 weeks*<br>**Activity level reduction**<br>*+ reduction reported in past 1 week*<br>*++ reduction reported in past 1-4 weeks*<br>*+++ reduction reported for > 4 weeks*<br>**Respiratory sign/s Y/N**<br>*Cough, wheeze, stertor, stridor, increased respiratory effort reported in past 3 months*<br>**Lymph node Y/N**<br>*Superficial LN/s reported to be visibly enlarged*<br>**Non-healing wound visible? Y/N**	+ or Y on any 1 or 2 clinical signs	++ for any single clinical sign<br>OR<br>+ and/or Y on ≥ 3 clinical signs	+++ for any clinical sign<br>OR<br>++ and/or Y for ≥ 2 clinical signs
**Lung postmortem finding**<br>*1: ≤ 5 focal lesions, each < 10mm diameter*<br>*2: >5 focal lesions, or focal lesions >10mm diameter and confined to one lobe*<br>*3: focal lesions >10mm seen in >1 lobe*<br>*4: diffuse change confined to <50% of one or two lobes only*<br>*5: diffused change to >2 lobes or to >50% of one or two lobes*	1	2 or 3	4 or 5
**Thoracic lymph nodes postmortem finding**<br>*Measurement in any one axis*	Up to 30 mm	30 – 75 mm	>75 mm
**Pleural fluid postmortem finding**	Mild increase noted and/or<br><100 ml volume	Moderate increase noted, and/or<br>100 – 500 ml volume	Marked increase noted and/or<br>>500 ml volume

Y, yes; N, no.
