# Supplementary material for: Comparative thoracic radiography in healthy and tuberculosis-positive sun bears (Helarctos malayanus)
Source: Front Vet Sci. 2025 Jan 6;11:1460140. doi: 10.3389/fvets.2024.1460140 (PMC11743561; doi:10.3389/fvets.2024.1460140)
Supplement: Supplementary file 2 [file Table_2.pdf]

Supplementary Table 2. Clinical signs and postmortem findings grades from 23 sun bears (*Helarctos malayanus*) at a sanctuary in Cambodia with culture - confirmed tuberculosis, along with radiologist reported features reported from thoracic radiographs.

**Study ID**	**Clinical signs**	**Postmortem findings**			**Reader**	**Radiographic features**								
		Lung	*LN*	*Pleural fluid*		Bronchial pattern	Broncho-interstitial pattern	Interstitial pattern	Interstitial to alveolar pattern	Alveolar pattern	Nodular pattern	LN	Pleural fluid	**Abnormal study**
SB099	-	-	CM+ TB++	-	R1		+							
					R2		+							
SB061*	+	-	-	-	R1	+								
					R2	+								
SB027	+	-	CM++ TB+++	-	R1							+		yes
					R2	+						+		yes
SB038	++	-	CM+	-	R1		+							
					R2		+	+						
SB084	-	+	-	-	R1		+							
					R2		+							
SB034	-	+	CM+ TB+	+	R1									
					R2		+							
SB098	+	++	TB+	-	R1		+							
					R2	+								
SB013	-	++	CM+	-	R1					+				
					R2	+								
SB058	+	++	CM++	-	R1						+			yes
					R2						+			yes
SB088	-	++	-	+	R1	+				+			+	yes
					R2				+					yes
SB090	+	++	-	-	R1		+							
					R2	+								
SB006	+++	++	CM++ TB+++	++	R1		+	+				+	+	yes
					R2			+			+	+		yes

Supplementary Table 2. (continued)

**Study ID**	**Clinical signs**	**Postmortem findings**			**Reader**	**Radiographic features**								
		Lung	*LN*	*Pleural fluid*		Bronchial pattern	Broncho-interstitial pattern	Interstitial pattern	Interstitial to alveolar pattern	Alveolar pattern	Nodular pattern	LN	Pleural fluid	**Abnormal study**
SB049	+++	++	CM++<br>TB+++	-	R1					+		+		yes
					R2		+					+		yes
SB105	++	+++	CM++<br>TB+	+	R1	+								yes
					R2	+								
SB106	++	+++	CM++	+	R1		+			+				
					R2	+								
SB059	++	+++	CM++<br>TB++	+	R1	+		+					+	yes
					R2		+		+					yes
SB025	+++	+++	-	-	R1	+				+				yes
					R2	+				+				yes
SB079	+++	+++	-	++	R1	+							+	yes
					R2		+			+				
SB096	+++	+++	CM++<br>TB+	+	R1	+			+	+				yes
					R2				+					yes
SB015	+++	+++	CM++	+++	R1		+				+		+	yes
					R2			+			+			yes
SB042	+++	+++	CM++<br>TB+++	++	R1					+			+	yes
					R2					+		+		yes
SB066	+++	+++	CM+<br>TB+	+	R1				+	+				yes
					R2				+					yes
SB083	+++	+++	CM+<br>TB+	-	R1	+			+					yes
					R2			+		+				yes

**Mycobacterium tuberculosis* cultured from a non-healing wound and mesenteric lymph node
LN, lymph node; CM, cranial mediastinal; TB, tracheobronchial; R1, radiologist 1; R2, radiologist 2
